# Cirrhosis-induced defects in innate pulmonary defenses against *Streptococcus pneumoniae*

**DOI:** 10.1186/1471-2180-7-94

**Published:** 2007-10-23

**Authors:** Katie L Propst-Graham, Laurel C Preheim, Elizabeth A Vander Top, Mary U Snitily, Martha J Gentry-Nielsen

**Affiliations:** 1 Department of Medical Microbiology and Immunology, Creighton University School of Medicine, Omaha, Nebraska, USA; 2 Research Service, Omaha Veterans Affairs Medical Center, Omaha, Nebraska, USA; 3 Department of Medicine, University of Nebraska College of Medicine, Omaha, Nebraska, USA

## Abstract

**Background:**

The risk of mortality from pneumonia caused by *Streptococcus pneumoniae *is increased in patients with cirrhosis. However, the specific pneumococcal virulence factors and host immune defects responsible for this finding have not been clearly established. This study used a cirrhotic rat model of pneumococcal pneumonia to identify defect(s) in innate pulmonary defenses in the cirrhotic host and to determine the impact of the pneumococcal toxin pneumolysin on these defenses in the setting of severe cirrhosis.

**Results:**

No cirrhosis-associated defects in mucociliary clearance of pneumococci were found in these studies, but early intrapulmonary killing of the organisms before the arrival of neutrophils was significantly impaired. This defect was exacerbated by pneumolysin production in cirrhotic but not in control rats. Neutrophil-mediated killing of a particularly virulent type 3 pneumococcal strain also was significantly diminished within the lungs of cirrhotic rats with ascites. Levels of lysozyme and complement component C3 were both significantly reduced in bronchoalveolar lavage fluid from cirrhotic rats. Finally, complement deposition was reduced on the surface of pneumococci recovered from the lungs of cirrhotic rats in comparison to organisms recovered from the lungs of control animals.

**Conclusion:**

Increased mortality from pneumococcal pneumonia in this cirrhotic host is related to defects in both early pre-neutrophil- and later neutrophil-mediated pulmonary killing of the organisms. The fact that pneumolysin production impaired pre-neutrophil-mediated pneumococcal killing in cirrhotic but not control rats suggests that pneumolysin may be particularly detrimental to this defense mechanism in the severely cirrhotic host. The decrease in neutrophil-mediated killing of pneumococci within the lungs of the cirrhotic host is related to insufficient deposition of host proteins such as complement C3 on their surfaces. Pneumolysin likely plays a role in complement consumption within the lungs. Our studies, however, were unable to determine whether pneumolysin more negatively impacted this defense mechanism in cirrhotic than in control rats. These findings contribute to our understanding of the defects in innate pulmonary defenses that lead to increased mortality from pneumococcal pneumonia in the severely cirrhotic host. They also suggest that pneumolysin may be a particularly potent pneumococcal virulence factor in the setting of cirrhosis.

## Background

Pneumonia is among the top ten causes of death in the United States and the most common cause of death due to infection [1,2]. The Gram positive bacterium *Streptococcus pneumoniae *is the leading cause of community-acquired pneumonia [3], and is an increasing concern due to the emergence of multi-antibiotic resistant strains [4,5]. Certain patient populations are at a particularly increased risk for pneumococcal infection, including young children, the elderly, and patients with immunodeficiencies or other medical conditions such as cirrhosis [6-9]. Cirrhosis is one of the most common causes of acquired immunodeficiency, and it is well known that mortality from pneumococcal pneumonia is very high in cirrhotics, even with appropriate intensive care support and antibiotic treatment [10-12]. In one particular multi-center study, cirrhosis of the liver increased the risk of death from invasive pneumococcal pneumonia more than any other condition analyzed [11]. However, the specific defect(s) in host defense responsible for this increased morbidity and mortality have not been clearly elucidated. In order to study the interaction between *S. pneumoniae *and innate host defenses in the cirrhotic host, our laboratory was the first to develop a cirrhotic rat model of pneumococcal pneumonia [13].

The progression of pneumococcal pneumonia requires that the organism evade a wide array of innate pulmonary host defenses. Once *S. pneumoniae *colonizes the airways, the mucociliary clearance apparatus, composed of mucus entrapment and the beating of ciliated epithelial cells lining the bronchi, prevents pneumococci from moving down into the lower respiratory tract. If pneumococci evade this host defense and enter the lungs, they become deposited on the alveolar lining layer. This thin aqueous layer covering the surface of the pulmonary epithelium contains numerous proteins and peptides possessing antibacterial activity. Lysozyme, lactoferrin, β-defensins, and surfactant within alveolar lining fluid constitute an important immediate defense against pneumococci prior to the recruitment of neutrophils [14-16]. Resident alveolar macrophages also phagocytose and kill many types of organisms that escape mucociliary clearance. However, previous research has shown that alveolar macrophages do not efficiently phagocytose and kill highly encapsulated type 3 *S. pneumoniae *[17-19]. For example, we showed that only 11% of alveolar macrophages from the lungs of either cirrhotic or control rats infected with type 3 *S. pneumoniae *had pneumococci associated with them [20]. The inability of macrophages to kill this organism was further confirmed in a murine model of pneumococcal pneumonia in which alveolar macrophage depletion failed to reduce pneumococcal clearance from the lungs [21]. Recruited pulmonary neutrophils are an important host defense against pneumococci. However, for phagocytosis and efficient neutrophil-mediated defense against *S. pneumoniae*, the organisms must be opsonized, notably with fragments of complement component C3 [22,23].

Pneumococci possess multiple virulence factors that facilitate evasion of host defenses [24,25]. One such virulence factor, pneumolysin, is a 53-kDa protein made by all clinical *S. pneumoniae *isolates. Pneumolysin is a pore-forming toxin that can be released from lysed or intact pneumococci during late log phase [26]. Pneumolysin has both cytotoxic and complement-activating activities [27,28]. The cytotoxic activity affects a wide array of host cells and contributes to lung damage during the early stages of pneumococcal pneumonia [29-31]. The complement-activating activity activates and consumes complement proteins [32], thus diverting complement away from the bacterial surface. This activity prevents efficient opsonophagocytosis and killing by host phagocytes [33].

Our laboratory has previously shown that, like their human counterparts, cirrhotic rats with ascites are hypocomplementemic [34] and are more susceptible to pneumococcal infection. They have increased mortality compared to control animals following both pulmonary and intravenous pneumococcal challenges with type 3 *S. pneumoniae *[13,34]. In addition, we have shown that the complement-activating activity of pneumolysin is particularly detrimental to cirrhotic rats following an intravenous challenge [34]. We therefore hypothesized that the severely cirrhotic host has increased mortality from pneumococcal pneumonia due to compromised innate defenses, and pneumolysin may also be especially detrimental to the cirrhotic rats during a respiratory tract infection. To test this hypothesis, we examined several innate pulmonary anti-pneumococcal defenses and the impact of pneumolysin in cirrhotic vs. control rats following pulmonary challenge. Mucociliary clearance, early (pre-neutrophil recruitment) and later (neutrophil-mediated) defenses, and complement deposition on pneumococci within the rats' lungs were quantified. The effects of pneumolysin production by the pneumococci were analyzed using isogenic mutant pneumococcal strains differing in their ability to produce pneumolysin.

## Results

### Mucociliary Clearance Is Not Impaired in Cirrhotic Rats

**Figure 1:**
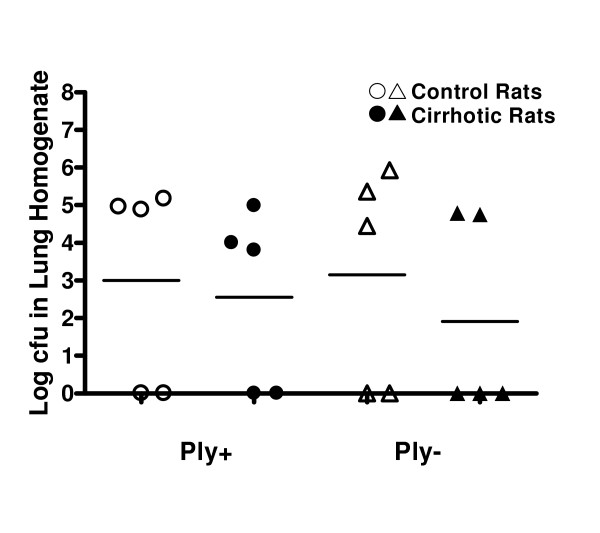
Total number of pneumococci recovered from individual rats' lungs five hours after an intranasal challenge with 1 × 10^8 ^cfu of pneumolysin sufficient (Ply+) or pneumolysin deficient (Ply-) *S. pneumoniae*. Horizontal bars represent the mean values for each group.

To determine whether mucociliary clearance is impaired in cirrhotic rats, the beat frequency of tracheal cilia and pneumococcal movement from the nasopharynx into the lungs were quantified. To examine the impact of pneumolysin production on mucociliary clearance, these experiments were performed with isogenic type 3 WU2-derived pneumolysin-producing (Ply+) and pneumolysin-deficient (Ply-) *S. pneumoniae *strains. Cirrhotic and control rats were infected intranasally with 1 × 10^8 ^colony forming units (cfu) of either the Ply+ or the Ply- strain. The numbers of pneumococci reaching the rats' lungs and the beat frequency of cilia on their tracheal epithelial cells then were quantified 5 hours post-infection. There were no differences between cirrhotic and control rats in the number of pneumococci recovered from their lungs after infection with either the Ply+ or Ply- strain (Figure 1). There also were no differences between the numbers of Ply+ vs. Ply- pneumococci isolated from the lungs of either group of rats. Consistent with the equivalent movement of the two pneumococcal strains into the lungs of cirrhotic and control rats, the beat frequency of cilia on their tracheal epithelial cells was comparable 5 h after an intranasal challenge with 1 × 10^8 ^cfu of either the Ply+ strain (8.4 ± 0.3 Hz vs. 8.0 ± 0.5 Hz, respectively) or the Ply- strain (9.2 ± 1.0 Hz vs. 8.7 ± 0.8 Hz, respectively).

### Cirrhosis Impairs Pre-Neutrophil-Mediated Pneumococcal Killing within the Lungs

**Figure 2:**
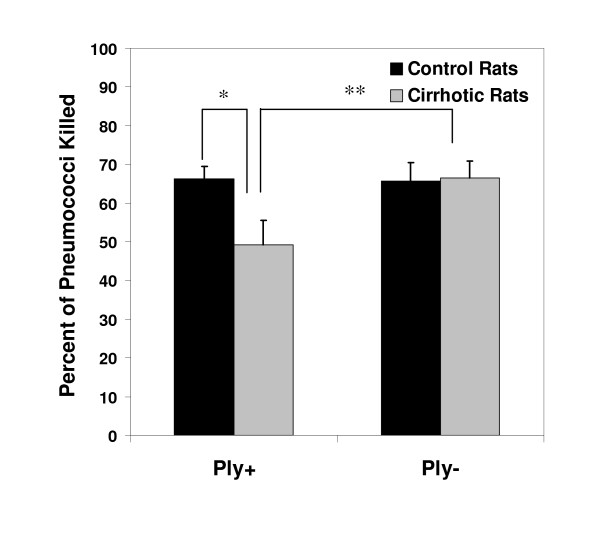
Pre-neutrophil killing of Ply+ and Ply- *S. pneumoniae *within the lungs of cirrhotic and control rats (n = 8–9) measured exactly 1 hour after a transtracheal challenge with 1 × 10^6 ^cfu. The cirrhotic rats killed significantly fewer Ply+ than control rats (* *p *= 0.035). Cirrhotic rats also killed significantly fewer of the Ply+ than Ply- organisms (** *p *= 0.038).

To determine whether early (pre-neutrophil-mediated) defenses were impaired in the lungs of the cirrhotic host, *in vivo *pneumococcal killing assays were conducted within the rats' lungs. These experiments were performed with the WU2-derived Ply+ and Ply- mutants to examine whether pneumolysin production protects *S. pneumoniae *from these early pulmonary defenses. The rats were infected transtracheally with 1 × 10^6 ^cfu of Ply+ or Ply- and then sacrificed exactly 1 hour later, before significant numbers of neutrophils were detectable in their lungs. Plate counts of the rats' lung homogenates showed a significant decrease (*p *= 0.035) in pre-neutrophil-mediated killing of the Ply+ strain in cirrhotic compared to control rats (Figure 2). By contrast, there was no difference in pre-neutrophil-mediated killing by the two groups of rats when the Ply- strain was used. Furthermore, killing of the Ply+ and Ply- strains was equivalent in the control rats, whereas killing of the Ply+ strain was significantly lower (*p *= 0.038) than that of the Ply- strain in cirrhotic animals. This suggests that pneumolysin selectively impairs pre-neutrophil defenses in the cirrhotic host with ascites.

To help elucidate what antibacterial components of pre-neutrophil defenses were defective within the lungs of the cirrhotic rats, anti-pneumococcal factors including lysozyme, lactoferrin, and complement component C3 were quantified in bronchoalveolar lavage fluid. All values were determined for uninfected cirrhotic and control rats using enzyme activity or enzyme linked immunosorbent assays. Mean lysozyme activity was significantly decreased in the cirrhotic animals compared to controls (1448 U/ml vs. 1687 U/ml; *p *= 0.028), whereas levels of lactoferrin were similar in cirrhotic and control rats (data not shown). Complement C3 levels in the lavage fluid from cirrhotic rats also were significantly decreased to less than half of those in control animals (1185 ng/ml vs. 2443 ng/ml; *p *= 0.012).

### Cirrhosis Diminishes Neutrophil-Mediated Killing within the Lungs

**Figure 3:**
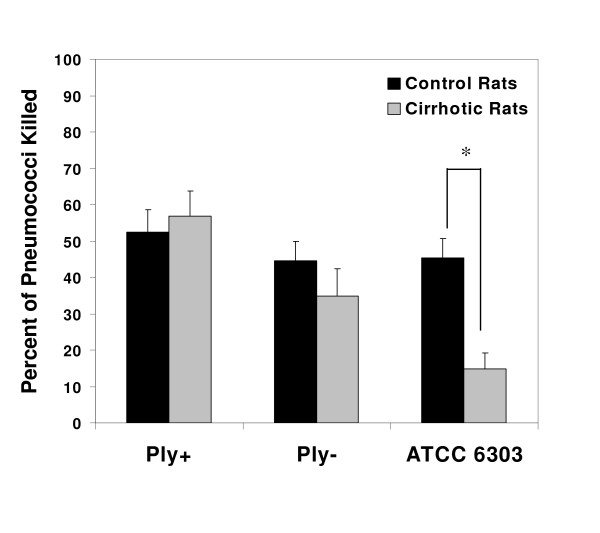
Neutrophil-mediated killing of three *S. pneumoniae *strains within the lungs of cirrhotic and control rats (n = 5–6). After a 5 hour LPS-induced neutrophil recruitment period, rats were challenged transtracheally with 1 × 10^6 ^of *S. pneumoniae *and killing was quantified exactly 1 hour later. Significantly fewer ATCC 6303 organisms were killed by cirrhotic rats than by control rats (* *p *= .001).

*In vivo *experiments quantifying neutrophil-mediatedpneumococcal killing were conducted by pre-recruiting neutrophils into the rats' lungs with lipopolysaccharide. Our laboratory had previously shown that neutrophil phagocytosis and killing of *S. pneumoniae *ATCC 6303 are decreased within the lungs of cirrhotic rats [19,20]. To determine the impact of pneumolysin on neutrophil-mediated killing, these experiments were conducted using the Ply+ and Ply- pneumococcal mutants. Five hours after recruitment of neutrophils into the lungs there was no reduction in intrapulmonary killing of either the Ply+ or Ply- organisms in cirrhotic animals compared to controls (Figure 3). In addition, the ability of the WU-derived pneumococci to produce pneumolysin did not affect neutrophil-mediated killing in either group of animals. Because these results differed from our previous findings, we repeated the assay with *S. pneumoniae *ATCC 6303. Pulmonary killing of this strain was significantly decreased (*p *= 0.001) in the cirrhotic rats, confirming our previous findings (Figure 3).

### Cirrhosis Diminishes Deposition of Complement C3 on Pneumococci

**Figure 4:**
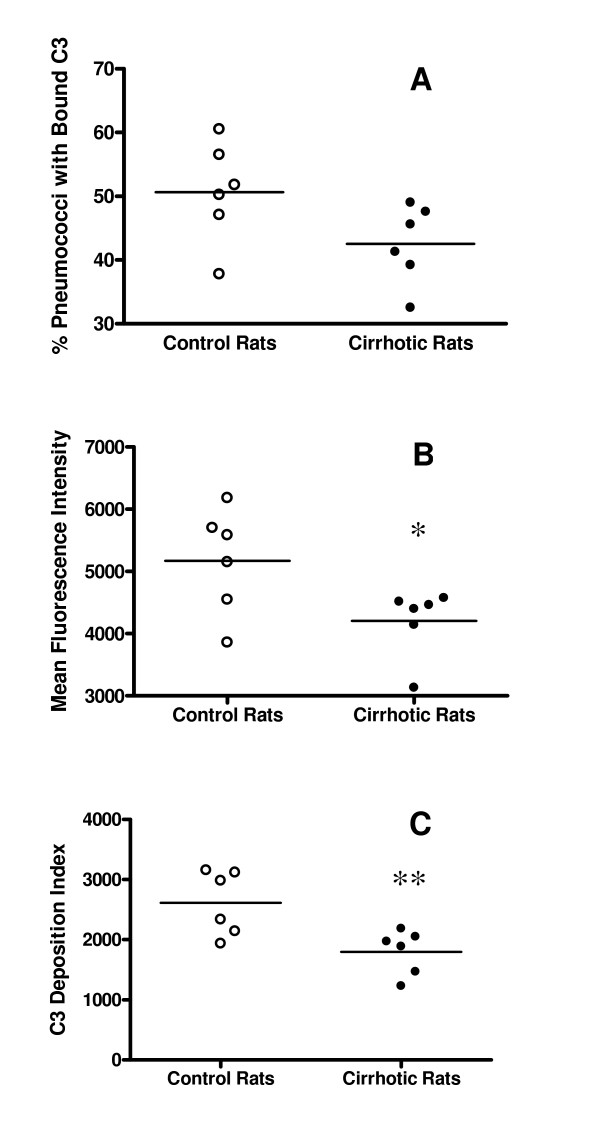
Quantification of complement deposition on pneumococci recovered from the lungs of cirrhotic and control rats 15 min after infection with 1 × 10^9 ^cfu of *S. pneumoniae *ATCC 6303. Flow cytometry was used to determine: (A) – percentage of pneumococci positive for bound C3; (B) – mean fluorescent intensity of pneumococci with bound C3; and (C) – deposition index calculated by multiplying the percentage of pneumococci positive for C3 by their fluorescent intensity. Both the mean fluorescent intensity (*: *p *= 0.04) and deposition index (**: *p *= 0.01) were significantly lower for organisms recovered from the lungs of cirrhotic vs. control rats.

To determine whether reduced pulmonary C3 levels in the cirrhotic rats corresponded to reduced complement-mediated opsonization of pneumococci within their lungs, we quantified C3 deposition on pneumococci following infection. Cirrhotic and control rats were infected transtracheally with 1 × 10^9 ^cfu of *S. pneumoniae *ATCC 6303. Exactly 15 minutes later, pneumococci recovered from the lungs of each rat by lavage were treated *in vitro *with a FITC-conjugated anti-C3 antibody. Flow cytometry then was used to quantify C3 deposition on the surface of the bacteria. The mean percentage of pneumococci with bound C3 was lower in the cirrhotic rats than in controls (Figure 4A), but the difference was not statistically significant (*p *= 0.075). However, the mean fluorescent intensity of pneumococci recovered from the lungs was significantly decreased (*p *= 0.04) in the cirrhotic rats (Figure 4B), as was a deposition index calculated by multiplying the percent of pneumococci positive for C3 by the fluorescent intensity (Figure 4C; *p *= 0.01).

## Discussion

Although mucociliary defects previously have not been linked to cirrhosis, we studied this innate defense because of its importance in preventing nasal pneumococcal colonization from progressing to a lower respiratory tract infection. Previous research performed *in vitro *has indicated that pneumolysin can negatively impact mucociliary clearance [35,36]. To identify potential cirrhosis-induced deficits in clearance and examine the effects of pneumolysin on this host defense mechanism in the cirrhotic host, the present studies were conducted with WU2-derived type 3 pneumococcal strains differing only in their ability to produce pneumolysin. There were no differences between cirrhotic and control rats in either pneumococcal movement from the nasopharynx to the lungs or ciliary beat frequency. Pneumolysin production also failed to impact pneumococcal movement into the lungs or mucociliary beating in either group of rats. Although it is possible that a more pronounced pneumolysin-induced effect would have been found over a longer post-infection period, pneumolysin does not appear to preferentially damage this host defense mechanism in the cirrhotic rats.

The remainder of the studies focused on innate host defenses taking place within the lungs themselves. These defenses were analyzed both before and after recruitment of neutrophils into the rats' lungs by instillation of lipopolysaccharide. To differentiate these two conditions they have been designated as pre-neutrophil-mediated (occurring before neutrophil recruitment) and neutrophil-mediated (occurring after recruitment of neutrophils into the lungs). Pre-neutrophil-mediated killing of the Ply+ pneumococcal strain was significantly reduced in cirrhotic rats compared to controls, whereas killing of the Ply- strain was equivalent in the two groups of animals. Moreover, the ability of the organisms to produce pneumolysin provided a significant protective advantage against pre-neutrophil-mediated killing in the lungs of cirrhotic but not control rats. Taken together, these results indicate that pre-neutrophil-mediated killing of pneumococci is diminished in the lungs of our cirrhotic rats, and that pneumolysin contributes more profoundly to pneumococcal resistance against this innate defense in the setting of cirrhosis. Pneumolysin has been shown to damage pulmonary cells, disrupt lung integrity, and decrease phagocyte function [29,32,36-41], all of which are detrimental to pulmonary defenses. Damage to the pulmonary epithelium and endothelium also may be more pronounced in the cirrhotic rats due to reduced macrophage activity or reduced levels of certain antibacterial factors even prior to infection.

Although a number of previous studies have quantified antibacterial components of alveolar lining fluid, such as lactoferrin, lysozyme, complement, defensins, and surfactant [14,15,42-45], to our knowledge they have not been performed in cirrhotic hosts. Therefore, to help elucidate which types of pulmonary antibacterial components are altered in the cirrhotic rats three anti-pneumococcal factors were quantified in lavage fluid from uninfected cirrhotic and control rats. Lysozyme activity was significantly lower in the bronchoalveolar lavage fluid of cirrhotic rats compared to their controls. To our knowledge, this is the first report of a link between liver damage and decreased lysozyme in lung lavage fluid. Because lysozyme breaks down peptidoglycan in the pneumococcal cell wall [42], lowered lysozyme activity would help explain the diminished pre-neutrophil-mediated killing of pneumococci in the cirrhotic rats.

Our laboratory and others have shown that cirrhotic hosts are often hypocomplementemic, with decreased levels of complement component C3 in their serum and ascites fluid [13,46,47]. The present study demonstrated that C3 levels are also significantly reduced in cirrhotic rats' bronchoalveolar lavage fluid. This deficiency could negatively impact innate pulmonary defenses in several ways. First, it would directly decrease the opsonizing capacity of the alveolar lining fluid since C3-derived complement components are major opsonins for *S. pneumoniae *[48]. Secondly, by reducing overall inflammation in the lungs it would blunt the influx of bactericidal factors from serum. Finally, reduced pulmonary complement levels would make the cirrhotic host particularly vulnerable to complement depletion by the complement-activating activity of pneumolysin. This would be consistent with previous work from our laboratory using an intravenous pneumococcal infection model, in which the complement-activating activity of pneumolysin was especially detrimental for cirrhotic rats [34]. Unlike control animals, the cirrhotic animals could not adequately replenish serum complement that was consumed by pneumolysin during the bacteremic infection [46].

Because an antibody to rat lactoferrin is not commercially available, lactoferrin in the rats' lavage fluid was quantified using a kit designed for human samples. The lactoferrin levels determined for the two groups of rats were equivalent, but this result must be interpreted with caution because the levels in all of the rats were under 5 ng/ml. This was most likely due to poor cross-species reactivity of the antibodies contained in the kit. Because lactoferrin has been shown to be directly bactericidal for pneumococci [49], it will be important for this assay to be repeated in the future with a rat-specific anti-lactoferrin antibody when one becomes available.

Pulmonary neutrophil-mediated defenses also were compared in cirrhotic and control rats by pre-recruiting neutrophils into the rats' lungs before instillation of the pneumococci. We used lipopolysaccharide rather than lipoteichoic acid for this purpose because we previously demonstrated that lipopolysaccharide has the advantage of inactivating confounding pre-neutrophil-mediated anti-pneumococcal factors [50]. This allows the differentiation of neutrophil-mediated killing from non-neutrophil-mediated killing. Consistent with our previous findings [19,20], we observed a reduction in neutrophil-mediated killing of *S. pneumoniae *ATCC 6303 in the cirrhotic animals. However, there was no difference in killing between the two groups of animals when they were challenged with either the Ply+ or Ply- mutants derived from the WU2 pneumococcal strain. These results may be explained by inherent differences between the ATCC 6303 and WU2-derived strains. For example, unpublished studies in our laboratory have shown that even large numbers of WU2-derived pneumococci injected by the transtracheal route fail to cause mortality in our rats. This indicates that the virulence of pneumococci from this genetic background is intrinsically inferior to that of ATCC 6303 in our rat model. Even though ATCC 6303 and WU2 are of the same capsule type, they produce PspA's from completely different clades. Because PspA has been shown to decrease complement activation at the pneumococcal surface [51], it is possible that differences in PspA type could help explain the greater difficulty our hypocomplementemic rats have killing ATCC 6303 within their lungs than the WU2-derived strains. Given the limited virulence of the WU2 strains in our rats, it is difficult to use them to determine whether pneumolysin production impairs pulmonary neutrophil-mediated killing within the lungs. The use of isogenic Ply+ and Ply- mutants derived from ATCC 6303 would be ideal for answering this question, but our attempts to procure these genetically modified strains in the 6303 background have been unsuccessful to date.

Our laboratory has previously examined the mechanism(s) for decreased neutrophil-mediated killing of *S. pneumoniae *ATCC 6303 in cirrhotic rats. Using *in vivo *assays, we demonstrated that adequate numbers of neutrophils are recruited into the lungs of cirrhotic rats following a transtracheal infection [52]. However, the cirrhotic rats' neutrophils are significantly impaired in their ability to take up the pneumococci when phagocytosis is measured inside the rats' lungs [19,20]. By contrast, neutrophils isolated from the peripheral blood of cirrhotic rats and tested *in vitro *phagocytosed serum-opsonized pneumococci as efficiently as neutrophils from control animals [19]. This indicated that decreased pulmonary neutrophil phagocytosis in the cirrhotic rats was not due to inherent defects in the neutrophils themselves but was more likely due to ineffective opsonization of the organisms within the cirrhotic rats' lungs. This argument was strengthened by the present study demonstrating both significantly decreased levels of complement component C3 in the alveolar lining fluid of cirrhotic rats and decreased complement deposition on the surface of pneumococci recovered from their lungs after a transtracheal infection. These data provide a potential mechanism to explain the reduced neutrophil-mediated killing of pneumococci and help explain the impaired clearance of *S. pneumoniae *from the lungs of this host with severe cirrhosis.

## Conclusion

In our rat model of carbon tetrachloride-induced cirrhosis, mucociliary clearance appears to be intact, but cirrhotic rats have significant impairments in both pre-neutrophil-mediated and neutrophil-mediated innate pulmonary defenses against *S. pneumoniae*. The defect in the early bactericidal activity of alveolar lining components is related, at least in part, to significantly reduced levels of the antibacterial proteins lysozyme and complement C3 in the cirrhotic rats. The impairment of neutrophil-mediated killing appears to be related to decreased deposition of complement on the organisms within the lungs of the cirrhotic rats. The contribution of pneumolysin production to resistance against pre-neutrophil-mediated killing was more pronounced during infection of cirrhotic vs. control rats. Taken together, these data indicate numerous defects in innate pulmonary defenses that help explain the increased susceptibility of the severely cirrhotic host to pneumococcal pneumonia. In conjunction with earlier work from our laboratory they also indicate that consumption of complement by pneumolysin may be particularly detrimental to pulmonary host defenses in a hypocomplementemic cirrhotic host.

## Methods

### Animals

Outbred 200 gram male Sprague-Dawley rats (Charles River, Kingston, NY) were housed in solid bottomed cages with a 12 hour light/dark cycle. They were fed standard rat chow (Kenwod Feeds, Omaha, NE) *ad libitum *and tap water containing 1.0 mM phenobarbital (Sigma Chemical, St Louis, MO) for stimulation of the P4502E1 cytochrome system. Cirrhosis was induced by weekly gavage with the hepatotoxin carbon tetrachloride (CCl_4_) as described previously [13]. Briefly, rats under light isoflurane anesthesia (Minrad, Inc., Bethlehem, PA) were gavaged weekly for 8–16 weeks with graded doses of CCl_4 _(Sigma). Two days post-gavage the animals' weight changes were used to calculate the following week's dose. All rats used for experimentation were rested from gavage at least 1 week, had a stable accumulation of abdominal ascites fluid for at least 2 consecutive weeks, and had cirrhotic livers upon dissection. Age-matched control rats were fed rat chow and water also containing 1.0 mM phenobarbital. They were weighed and gavaged weekly under anesthesia with equivalent doses of phosphate buffered saline (PBS) for the same number of weeks. In each of the experiments, the rats were sacrificed by a 75 mg/kg intraperitoneal injection of pentobarbital (Nembutal; Abbott Laboratories, Chicago, IL) followed by exsanguination. All experimental procedures were approved by the Institutional Animal Care and Use Subcommittee at the Omaha Veterans Affairs Medical Center.

### Bacterial Strains and Growth Conditions

Three *S. pneumoniae *strains were used. ATCC 6303 (American Type Culture Collection, Rockville, MD) is a clinical isolate with a large type 3 capsule that belongs to PspA clade 5. For determination of the contribution of pneumolysin to virulence, certain experiments utilized isogenic mutants derived from the WU2 strain of *S. pneumoniae *differing only in their ability to produce pneumolysin (kindly provided by M. K. Johnson, Tulane University School of Medicine, New Orleans, LA). WU2 is also a clinical pneumococcal strain that produces a type 3 capsule but, unlike ATCC 6303 belongs to PspA clade 2. WU2 strains are lethal to our rats when given intravenously [34] but not when infected transtracheally. The Ply+ and Ply- mutants were created from a spontaneously occurring rough (nonencapsulated) WU2 isolate (WU2R) as described previously [53,54]. Briefly, the gene for pneumolysin was excised from the chromosome and plasmids encoding a functional (Ply+) or a disrupted (Ply-) pneumolysin gene were transformed into WU2R. Further transformation with DNA from the smooth WU2 parent then was used to convert both WU2R strains back into the smooth, encapsulated form.

All *S. pneumoniae *strains were stored at -80°C in Todd-Hewitt broth containing 10% glycerol, and grown in Todd-Hewitt broth at 37°C and 5% CO_2_. For all experiments, the bacteria were collected by centrifugation, washed twice with PBS, and diluted to the appropriate optical density at 540 nm to achieve the desired inoculum for each experiment. The number of colony-forming units (cfu's) in the suspension then was determined retrospectively by serial dilution and plating on sheep blood agar plates (Remel, Lenexa, KS).

### Intranasal and Transtracheal Infections

Rats were infected intranasally with *S. pneumoniae *in a total of 100 μl of PBS under light isofluorane anesthesia (Midrad, Inc., Bethlehem, PA) for the mucociliary clearance studies. Following intranasal inoculation, the rats were held vertically for a few seconds to allow for aspiration of the fluid. Rats were infected transtracheally with *S. pneumoniae *under isofluorane anesthesia for the pre-neutrophil killing, neutrophil killing, and complement-deposition experiments as described previously [13]. Briefly, a small incision was made overlying the trachea, the trachea was made visible by blunt-end dissection, and a 22 gauge catheter (Becton, Dickinson and Co., Sandy, UT) was inserted into the trachea. The rat then was placed in a vertical position and the specified inoculum of *S. pneumoniae *suspended in 0.3 ml PBS was injected into the catheter. This was followed by 0.5 ml of air to simulate aspiration.

### Analysis of Mucociliary Clearance

Cirrhotic and control rats were infected intranasally with 1 × 10^8 ^cfu Ply+ or Ply- WU2 *S. pneumoniae *grown to late log phase. Five hours later, the animals were sacrificed and their lungs were removed *en bloc *and homogenized in a final volume of 10 ml ice cold PBS. Pneumococci reaching the lungs were quantified by standard plate counts of the serially diluted homogenized lung tissue. Baseline ciliary beat frequency was quantified on tracheal ring explants from the same rats using the Sisson-Ammons Analysis (SAVA) system as described previously [55]. Software by Ammons Engineering (Mt. Morris, MI) was used for whole field analysis. The frequency of the beating cilia was sampled in at least 6 different fields, and each animal's value was composed of the mean for 6 fields. This data then was compiled to produce average ciliary beat frequency in Hz for the two different groups of animals.

### *In vivo *Pneumococcal Killing Assays

To measure pulmonary killing of *S. pneumoniae *by pre-neutrophil-mediated defenses, cirrhotic and control rats were infected transtracheally with 1 × 10^6 ^cfu of the Ply+ or Ply- WU2-derived *S. pneumoniae *grown to mid-log phase. Exactly 1 hour post-infection each rat was sacrificed and its lungs were removed and homogenized as described above. Plate counts were performed on the homogenates, and killing of the pneumococci was quantified as the percent decrease in bacterial counts compared to the mean count obtained from two rats sacrificed immediately after infection (0 h) using the following formula:

*Percent Killing* = *(Lung cfu's at 0 h - Lung cfu's at 1 h)/(Lung cfu's at 0 h) *× *100*

To quantify killing of *S. pneumoniae *by pulmonary neutrophil-mediated defenses, neutrophils were first pre-recruited into the rats' lungs by transtracheal instillation of 20 μg of *Escherichia coli *O26:B6 lipopolysaccharide (Sigma, St. Louis, MO) suspended in 0.2 ml of PBS. Five hours after the instillation of LPS, the rats were infected transtracheally with 1 × 10^6 ^cfu of the ATCC 6303, the Ply+ WU2, or the Ply- WU2 strains of *S. pneumoniae *grown to mid-log phase. Exactly 1 hour post-infection each rat was sacrificed and its lungs were removed and homogenized. Neutrophil-mediated killing was quantified using the same formula shown above.

### Quantification of Pulmonary Anti-Pneumococcal Factors

Bactericidal factors were quantified in lavage fluid collected from cirrhotic and control rats by *in situ *bronchoalveolar lavage with a single 6 ml aliquot of ice cold PBS. Cells were removed from the lavage fluid by centrifugation and the supernatant was filter-sterilized and stored at -80°C until analyzed. Lysozyme activity in the lavage samples was quantified using the EnzChek^® ^Lysozyme Assay Kit (Molecular Probes, Eugene, OR). Lactoferrin was quantified using Bioxytech^® ^Lacto*f*-EIA for human lactoferrin (Oxis International, Inc., Portland, OR). Quantification of C3 was performed using a commercially available Rat C3 ELISA kit from Immunology Consultants Laboratory, Inc., Newberg, OR.

### In vivo Complement Deposition Assay

To quantify C3 deposition on the surface of pneumococci, cirrhotic and control rats were infected transtracheally with 1 × 10^9 ^cfu of *S. pneumoniae *ATCC 6303 grown to stationary phase. Exactly 15 minutes post-infection, each rat was sacrificed. The lungs were perfused with 30 ml of ice cold PBS, and then bronchoalveolar lavage was performed *ex vivo*. The lungs were washed with 10 ml aliquots of ice cold Hanks' Balanced Salt Solution without Mg++, Ca++ or phenol red (HBSS, Gibco/Invitrogen, Carlsbad, CA) that were collected by dependent drainage until a total volume of 50 ml was recovered. The rat pulmonary cells were removed from the lavage fluid by centrifugation at 450 × g for 30 minutes. The resulting supernatant was then centrifuged at 13,776 × g for 10 minutes to collect pneumococci recovered from the rats' lungs. Any remaining rat cells in the bacterial pellet were lysed twice by the addition of 10 ml distilled water followed by an equal volume of double-strength PBS. The final bacterial pellet was labeled by incubation at 37°C for 30 minutes with a 1:30 dilution of fluorescein-conjugated IgG fraction of goat anti-rat C3 antibody (Cooper Biomedical, Inc., Malvern, PA).

Following antibody labeling, the bacteria were washed in HBSS and fixed in PBS containing 1% formalin for flow cytometric analysis using a FACSAria flow cytometer (Becton Dickinson, San Jose, CA). Negative control samples consisted of the unlabeled bacteria used for rat infection and bacteria collected from each rat's lungs prior to antibody labeling. The positive control consisted of the bacterial suspension used for infection that was opsonized *in vitro *with normal rat serum for 30 minutes and then labeled with the anti-C3 antibody as described above. On each day of experimentation, a forward and side scatter plot of pneumococci grown in culture was used to set the analysis gate for pneumococci recovered from the rats' lungs. Histograms of count (number of events) vs. fluorescence at 530 nm were used to quantify C3 binding to the surface of the pneumococci.

### Statistical Analyses

All comparisons of values determined for cirrhotic vs. control rats or Ply+ vs. Ply- were made by Students *t *test. Prior to analysis, the data were first tested for normality and equal variance. If the data were non-parametric, the Mann-Whitney Rank Sum Test was used. For all tests, a *p *value <0.05 was considered significant. Error bars on all graphs represent standard errors of the means.

## Authors' contributions

This work was performed as part of the Master's thesis for KPG. KPG and MUS carried out all experimentation and aided the experimental design. EVT was instrumental in the development of the *in vivo *pneumococcal killing and complement deposition assays. LCP and MGN conceived the cirrhotic rat model and the overall experimental design of the study. KPG and MGN (thesis advisor) drafted the manuscript. All authors have read and approved the final manuscript.
